# Nutritional intake, biochemical profiles, and functional outcomes in elderly inpatients: A hospital-based cross-sectional study in Vietnam

**DOI:** 10.1371/journal.pgph.0006803

**Published:** 2026-07-16

**Authors:** Tran Thi Phuong Lan, Pham Van Phu, Le Thi Huong, Le Xuan Hung, Nguyen Quang Dung

**Affiliations:** 1 Department of Nutrition and Food Safety, School of Preventive Medicine and Public Health, Hanoi Medical University, Hanoi, Vietnam; 2 Department of Nutrition - C11, Military Hospital 354, Hanoi, Vietnam; 3 Department of Research Methodology and Biostatistics, School of Preventive Medicine and Public Health, Hanoi Medical University, Hanoi, Vietnam; African Population and Health Research Center, KENYA

## Abstract

Malnutrition poses a significant challenge for elderly inpatients, particularly in developing countries, contributing to adverse clinical outcomes. This cross-sectional study examined associations between nutritional intake, biochemical profiles, and functional outcomes in 264 elderly inpatients (mean age 73.39 ± 4.12 years, 50.4% male) at Military Hospital 354, Hanoi, Vietnam, from January to June 2025. Nutritional status was assessed using the Mini Nutritional Assessment Short Form (MNA-SF), in conjunction with energy intake (24-hour dietary recall), body mass index (BMI), serum albumin levels, Activities of Daily Living (ADL) scores, and EuroQol-5 Dimensions (EQ-5D) dimension scores (range: 5–15; summative approach used for simplicity in resource-constrained settings, though it may limit utility comparisons). Higher EQ-5D scores indicate worse health status. Results revealed 34.8% of participants were malnourished (MNA-SF < 8), 37.9% at risk (8–11), and 33.0% had hypoalbuminemia (<35 g/dL). Functional impairment (ADL < 6) affected 33.3%. Significant correlations were observed between MNA-SF and albumin (r = 0.44, p < 0.01) and ADL (r = 0.63, p < 0.01). Multiple linear regression, adjusted for age, sex, and comorbidities, revealed that MNA-SF and albumin were significantly associated with ADL (β = 0.43, p < 0.01; β = 0.13, p < 0.01) and EQ-5D (β = -0.46, p < 0.01; β = -0.12, p < 0.01). These findings underscore the urgent need for routine nutritional screening and interventions in Vietnamese hospitals to mitigate the impact of malnutrition on functional outcomes and quality of life. This study provides additional limited empirical evidence from Vietnam on the association between nutritional and functional measures among elderly inpatients, contributing to the geriatric nutrition literature in developing countries.

## Introduction

Malnutrition in hospitalized elderly patients is a major global health issue associated with adverse clinical outcomes such as prolonged hospital stays, increased readmissions, and elevated mortality [1,2]. Prevalence estimates range from 29% to 90%, driven by age-related physiological changes, comorbidities, and socioeconomic factors, with developing countries facing heightened challenges due to limited healthcare resources [1,3]. In Vietnam, a rapidly aging nation, malnutrition is particularly prevalent among elderly inpatients, with studies reporting rates as high as 72.2%, yet it often remains underdiagnosed and undertreated [4]. The Mini Nutritional Assessment Short Form (MNA-SF), a validated tool with 96% sensitivity and 98% specificity, is widely used to screen for malnutrition risk in elderly populations. Still, its adoption in resource-constrained settings, such as Vietnam, is limited [5].

Biochemical markers, notably serum Albumin, are critical for assessing nutritional status and predicting clinical outcomes in elderly inpatients [6]. Hypoalbuminemia (<35 g/dL), observed in up to 99% of hospitalized elderly, is strongly associated with increased mortality (relative risk: 4.1) and prolonged length of stay (relative risk: 5.2) [6]. Similarly, inadequate dietary energy intake, often below 1500 kcal/day, may exacerbate malnutrition and functional decline, particularly in socioeconomically disadvantaged settings [2]. Body Mass Index (BMI), a key anthropometric indicator, further reflects nutritional health, with low BMI (<19 kg/m²) indicating undernutrition and higher BMI (≥23 kg/m²) signaling metabolic risks in Asian populations [7]. Functional outcomes, as measured by the Activities of Daily Living (ADL) and EuroQol-5 Dimensions (EQ-5D), are closely tied to nutritional status, with malnourished patients exhibiting significant impairments in mobility, self-care, and quality of life [7,8]. Additionally, dysphagia, affecting 24.6% of elderly inpatients in Vietnam, increases malnutrition risk (odds ratio: 3.21), complicating nutritional management [8].

Despite these established links, research on malnutrition, biochemical profiles, and functional outcomes in elderly inpatients in developing countries, particularly Vietnam, remains limited [3,4]. Vietnam’s aging population faces unique challenges, including high chronic disease prevalence (e.g., hypertension, COPD) and socioeconomic factors such as low education and income, which elevate malnutrition risk (odds ratio: 1.83) [4,7]. While local studies highlight a high prevalence of malnutrition and its association with low Albumin, frailty, and reduced handgrip strength, comprehensive data on the interplay of nutritional intake, biochemical markers, and functional outcomes are scarce [4,8]. This gap hinders the development of targeted interventions to improve health outcomes in this vulnerable population. Mechanistically, malnutrition can exacerbate inflammation, leading to hypoalbuminemia and muscle wasting, which in turn impair functional outcomes such as mobility and self-care through pathways involving cytokine-mediated catabolism and reduced protein synthesis [9].

This study addresses these gaps by examining the relationships between nutritional intake, biochemical profiles, and functional outcomes in 264 elderly inpatients at a tertiary hospital in Vietnam. We aim to quantify the prevalence of malnutrition and its impact on functional status using validated tools (MNA, ADL, EQ-5D) and biochemical markers (Albumin). We also investigate how dietary energy intake and BMI, stratified by age, contribute to these outcomes, providing insights into age-specific nutritional risks. Using multiple linear regression models, we aim to identify key predictors of ADL and EQ-5D scores, while adjusting for age and sex, to inform clinical practice and policy in Vietnam and similar developing countries.

The primary objective is to investigate the associations between nutritional intake (MNA, energy intake), biochemical profiles (Albumin), and functional outcomes (ADL, EQ-5D) among elderly inpatients in Vietnam. Secondary objectives include assessing the prevalence of malnutrition and low BMI across age groups and determining their predictive roles in functional decline. This research contributes to the limited literature on geriatric nutrition in developing countries, providing evidence to inform nutritional interventions and improve health outcomes for hospitalized older adults.

## Materials and methods

### Study design and setting

This cross-sectional study was conducted at Military Hospital 354, a tertiary care facility in Hanoi, Vietnam, from January to June 2025. The hospital offers specialized care to a diverse population, including elderly patients with both chronic and acute conditions. The study investigated the associations between nutritional intake, biochemical profiles, and functional outcomes in elderly inpatients. Data on nutritional and functional assessments were collected prospectively, while demographic and clinical data were retrieved retrospectively from medical records. All data collection procedures were initiated only after approval was obtained from the Institutional Ethics Committee, ensuring compliance with ethical standards for research involving human subjects. The study was reported in accordance with the Strengthening the Reporting of Observational Studies in Epidemiology (STROBE) guidelines for cross-sectional studies (S1 Checklist).

### Participants

Eligible participants were inpatients aged 60 years or older admitted to the geriatric, internal medicine, or rehabilitation wards of Military Hospital 354. **Inclusion criteria included**: (1) hospital admission for ≥48 hours, (2) ability to provide informed consent or availability of a legal representative, and (3) complete data on nutritional and functional assessments. **Exclusion criteria were**: (1) terminal illness or palliative care status, (2) severe cognitive impairment (Mini-Mental State Examination score <10) precluding reliable responses, and (3) incomplete biochemical or anthropometric data. A convenience sample of 264 participants was recruited throughout the study. Convenience sampling was employed because consecutive enrollment of all eligible patients was not feasible due to ward-level scheduling and staffing constraints. A post hoc power analysis - conducted after data collection to verify sample adequacy rather than to justify the sampling design - confirmed that the achieved sample of 264 exceeds the minimum of 93 participants required to detect a moderate correlation (r = 0.30) with 80% power at α = 0.05 (two-tailed, accounting for 10% attrition), providing adequate statistical power for the planned correlation and regression analyses.

### Ethical considerations

The study was approved by the Ethics Committee of Hanoi Medical University (Permit: 1294/GCN-HMUIRR). Written informed consent was obtained from all participants or their legal representatives before enrollment. A signed Consent Form was obtained and securely filed for participants with potentially identifying data (e.g., demographic details).

### Data collection

Training dietitians and research nurses collected data within 48 hours of admission using standardized protocols at Military Hospital 354. The following assessments were performed:

### Nutritional assessment

- **Mini Nutritional Assessment Short Form (MNA-SF)**: Nutritional status was evaluated using the MNA-SF, a validated tool for elderly populations [5]. Scores range from 0 to 14 and are categorized as: 0–7 (malnourished), 8–11 (at risk), and 12–14 (normal). The MNA-SF includes questions about BMI, weight loss, appetite, and mobility.
- **Energy Intake**: Daily dietary intake was estimated using a 24-hour dietary recall conducted by trained dietitians via structured interviews that probed portion sizes and included hospital-provided meals, snacks, and supplements. The recall was cross-validated with ward records to minimize recall bias. Total energy (kcal/day) was calculated using Vietnamese food composition tables [10].
- **Body Mass Index (BMI)**: Weight and height were measured using a calibrated scale and stadiometer, with BMI (kg/m^2^) categorized as: < 19 (underweight), 19- < 21 (normal), 21- < 23 (normal), and ≥23 (overweight/obese), adapted for Asian populations [11].

### Biochemical assessment

- **Serum Albumin**: Blood samples were collected and analyzed for Albumin (g/dL) using a colorimetric assay at the hospital’s accredited laboratory. Hypoalbuminemia was defined as <35 g/dL [4]. Samples were processed within 24 hours of collection.

### Functional assessment

- **Activities of Daily Living (ADL)**: Functional status was assessed using a modified Katz ADL index, which evaluates seven activities (bathing, dressing, toileting, transferring, continence, feeding, and mobility) [12]. Each activity is scored as dependent (0) or independent (1), yielding a total score from 0 (complete dependence) to 7 (complete independence). The original six-item Katz scale was extended by adding a mobility item to capture better functional capacity among hospitalized elderly patients, consistent with clinical practice at Military Hospital 354. Functional impairment was defined as a modified Katz ADL score < 6.
- **EuroQol-5 Dimensions (EQ-5D)**: The EQ-5D-3L dimension scores were summed across five dimensions (mobility, self-care, usual activities, pain/discomfort, anxiety/depression), each scored from 1 (no problems) to 3 (extreme problems), yielding a total range of 5–15, with higher scores indicating more health problems [13]. A summative dimension score was used rather than a utility index because Vietnamese EQ-5D-3L value sets were not available at the time of data collection; the summative approach provides a transparent, assumption-free measure of cumulative problem burden suitable for within-sample comparisons in resource-constrained settings [14]. This approach departs from the standard EQ-5D-3L methodology, which derives health utility indices using country-specific tariffs; as a result, the scores reported here are not directly comparable to utility-weighted indices reported in the published literature. The construct validity of the summative scoring approach in the Vietnamese elderly population has not been established, and future studies should adopt utility-based scoring when country-specific value sets become available.

### Demographic and clinical data

- **Demographics**: Age, sex, education level, and cohabitation status were collected through structured interviews.
- **Clinical data**: Primary diagnoses (e.g., hypertension, COPD) and comorbidities were extracted from medical records using International Classification of Diseases (ICD-10) codes.

### Statistical analysis

Data were analyzed using R version 4.2.2 (R Foundation for Statistical Computing, Vienna, Austria). Descriptive statistics included means ± standard deviations for continuous variables (e.g., age, Albumin) and frequencies/percentages for categorical variables (e.g., MNA categories, sex). Normality was assessed using the Shapiro-Wilk test, which guided the choice of parametric or non-parametric tests.

- **Correlations**: Spearman’s rank correlation was used to examine associations between continuous variables (e.g., MNA vs. Albumin, Albumin vs. ADL), given non-normal distributions of ADL and EQ-5D scores.
- **Group comparisons**: Independent t-tests or Mann-Whitney U tests were used to compare continuous outcomes (e.g., ADL) between groups (e.g., MNA < 8 vs. 12–14, BMI < 19 vs. ≥ 23). Chi-square tests assessed differences in categorical outcomes (e.g., BMI categories by age group: 65–74 vs. ≥ 75).
- **Regression analysis**: Multiple linear regression models were fitted to identify predictors of ADL and EQ-5D scores, with independent variables including MNA, Albumin, BMI, energy intake, age, and sex. Models adjusted for confounders (age, sex, comorbidities) to estimate coefficients (β), 95% confidence intervals, and p-values. Multicollinearity was assessed using variance inflation factors (VIF). While bivariate Spearman correlations between MNA-SF, BMI, and energy intake were high (r = 0.72-0.77, Table 3), VIF values in the multiple regression models were MNA-SF: 2.48, BMI: 2.31, Albumin: 1.24, energy intake: 1.13, age: 1.06, and sex: 1.06, all well below the conventional threshold of 5 and below the more conservative threshold of 3 (maximum VIF = 2.48). High pairwise Spearman correlations do not necessarily translate into problematic multicollinearity in a multivariable model because VIF quantifies the proportion of a predictor’s variance that is linearly explained by all other predictors simultaneously, not by any single one. Tolerance values (1/VIF) ranged from 0.40 to 0.94, indicating that each predictor retained 40–94% unique variance after accounting for all others. The inclusion of additional covariates (age, sex, comorbidities) redistributes shared variance and attenuates the inflation that would be expected from bivariate correlations alone. Moreover, MNA-SF, BMI, and energy intake are conceptually independent dimensions of nutritional status - composite screening risk, anthropometric status, and actual dietary consumption, respectively - and their joint retention is warranted on substantive grounds despite moderate shared variance. Nonetheless, we acknowledge that the shared variance among these nutritional indicators warrants cautious interpretation of individual predictor coefficients, and we note that energy intake and BMI were non-significant in most models, consistent with collinearity-related attenuation. Model fit was assessed using R² and residual diagnostics.
- **Significance level**: A two-tailed p-value <0.05 was considered statistically significant.

## Results

### Participant characteristics

The study enrolled 264 elderly inpatients at Military Hospital 354, Hanoi, Vietnam, with a mean age of 73.39 ± 4.12 years. Of these, 50.4% (n = 133) were male, and 49.6% (n = 131) were female. The most common primary diagnoses were hypertension (13.6%, n = 36) and chronic obstructive pulmonary disease (8.7%, n = 23), with other conditions including diabetes and cardiovascular diseases. Detailed demographic and clinical characteristics are presented in Table 1.

**Table 1:** Demographic and clinical characteristics of elderly inpatients.

Characteristic	Value
**Age (years), mean ± SD**	73.39 ± 4.12
**Sex, n (%)**	
Male	133 (50.4%)
Female	131 (49.6%)
**Primary diagnosis, n (%)**	
Hypertension	36 (13.6%)
COPD	23 (8.7%)
Other	205 (77.7%)
**Education level, n (%)**	
Low (≤Secondary)	101 (38.3%)
High (>Secondary)	163 (61.7%)
**Cohabitation status, n (%)**	
Living alone	2 (0.8%)
Living with others	261 (98.9%)

### Nutritional status

Based on the Mini Nutritional Assessment Short Form (MNA-SF), 34.8% (n = 92) of participants were malnourished (score <8), 37.9% (n = 100) were at risk of malnutrition (score 8–11), and 25.4% (n = 67) had normal nutritional status (score 12–14). The mean MNA-SF score was 8.49 ± 3.75. Energy intake ranged from 950 to 2100 kcal/day, with no significant differences by sex (p = 0.12). BMI averaged 21.53 ± 2.96 kg/m², with 20.8% (n = 55) classified as underweight (<19), 22.0% (n = 58) as normal (19- < 21), 27.7% (n = 73) as normal (21- < 23), and 29.5% (n = 78) as overweight/obese (≥23). Nutritional and anthropometric data are summarized in Table 2.

**Table 2:** Nutritional, biochemical, and functional outcomes of elderly inpatients.

Characteristic	Value
**MNA-SF score, mean ± SD**	8.49 ± 3.75
MNA-SF category, n (%)*	
Malnourished (<8)	92 (34.8%)
At Risk (8–11)	100 (37.9%)
Normal (12–14)	67 (25.4%)
**Energy intake (kcal/day), mean ± SD**	1683.14 ± 174.88
**BMI (kg/m²), mean ± SD**	21.53 ± 2.96
**BMI category, n (%)**	
Underweight (<19)	55 (20.8%)
Normal (19- < 21)	58 (22.0%)
Normal (21- < 23)	73 (27.7%)
Overweight/Obese (≥23)	78 (29.5%)
**Serum albumin (g/dL), mean ± SD**	36.50 ± 3.75
**Albumin category, n (%)**	
Hypoalbuminemia (<35 g/dL)	87 (33.0%)
Normal (≥35 g/dL)	177 (67.0%)
**ADL score, mean ± SD**	5.17 ± 2.54
**ADL category, n (%)**	
Impaired (<6)	88 (33.3%)
Independent (6–7)	176 (66.7%)
**EQ-5D Score, mean ± SD**	
**Overall**	12.07 ± 2.55
Malnourished (MNA-SF < 8, n = 92)	13.91 ± 2.07
At risk (MNA-SF 8–11, n = 100)	12.0 ± 2.4
Normal (MNA-SF 12–14, n = 67)	10.5 ± 2.1

*Five participants had incomplete responses and were excluded from category-specific analysis; MNA-SF category percentages are based on n = 259 (malnourished: 92/259 = 35.5%, at risk: 100/259 = 38.6%, normal: 67/259 = 25.9%). Percentages reported in the text (34.8%, 37.9%, 25.4%) are based on the full sample (N = 264) for consistency with overall prevalence reporting

### Biochemical profiles

Serum Albumin levels averaged 36.50 ± 3.75 g/dL, with 33.0% (n = 87) of participants exhibiting hypoalbuminemia (<35 g/dL). Albumin levels were significantly lower in malnourished participants (MNA-SF < 8) compared to those with normal nutritional status (MNA-SF 12–14) (33.2 ± 3.9 vs. 37.8 ± 2.8 g/dL; t = -5.64, p < 0.001). No significant sex differences in Albumin levels were observed (p = 0.09). Biochemical data are presented in Table 2.

### Functional outcomes

The mean Activities of Daily Living (ADL) score was 5.17 ± 2.54, with 33.3% (n = 88) of participants exhibiting functional impairment (modified Katz ADL score < 6 out of 7). Malnourished participants had significantly lower ADL scores than those with normal nutritional status (3.2 ± 2.4 vs. 5.8 ± 1.7; U = 342, p < 0.001). The EuroQol-5 Dimensions (EQ-5D) dimension score indicated moderate impairment (mean 12.07 ± 2.55, range 5–15). Malnourished participants (MNA-SF < 8) had higher scores (13.91 ± 2.07, n = 92) compared to at-risk (12.0 ± 2.4, n = 100) and well-nourished participants (10.5 ± 2.1, n = 67) (U = 418, p < 0.001 for malnourished vs. well-nourished) (Table 2).

### Correlations

Spearman’s rank correlations revealed significant associations between nutritional and functional variables (Table 3). MNA-SF scores were positively correlated with Albumin (r = 0.44, p < 0.01) and ADL (r = 0.63, p < 0.01). Albumin levels were positively correlated with ADL scores (r = 0.41, p < 0.01) and negatively correlated with EQ-5D scores (r = -0.41, p < 0.01). BMI and energy intake showed weaker correlations with ADL (r = 0.32, p < 0.01; r = 0.47, p < 0.01) and EQ-5D (r = -0.31, p < 0.01; r = -0.49, p < 0.01).

**Table 3:** Spearman’s rank correlations between nutritional, biochemical, and functional variables in elderly inpatients.

Variable	MNA-SF	Albumin	BMI	Energy Intake	ADL	EQ-5D
**MNA-SF**	–	0.44**	0.72**	0.77**	0.63**	-0.57**
**Albumin**	0.44**	–	0.27**	0.32**	0.41**	-0.41**
**BMI**	0.72**	0.27**	–	0.74**	0.32**	-0.31**
**Energy Intake**	0.77**	0.32**	0.74**	–	0.47**	-0.49**
**ADL**	0.63**	0.41**	0.32**	0.47**	–	-0.61**
**EQ-5D**	-0.57**	-0.41**	-0.31**	-0.49**	-0.61**	–

Correlation coefficients (r) are presented, with significance indicated as: *p < 0.05, **p < 0.01. MNA-SF: Mini Nutritional Assessment Short Form; BMI: Body Mass Index; ADL: Activities of Daily Living; EQ-5D: EuroQol-5 Dimensions (higher scores indicate more health problems). Note: Significant correlations (r ≥ 0.40 or ≤ -0.40) are shaded for clarity in the table.

### Regression analyses

Multiple linear regression models, adjusted for age, sex, and comorbidities, identified predictors of ADL and EQ-5D scores (Table 4). For ADL, significant predictors included MNA-SF score (β = 0.43, 95% CI [0.34, 0.53], p = 0.000), Albumin (β = 0.13, 95% CI [0.07, 0.20], p = 0.000), Age (β = -0.07, 95% CI [-0.12, -0.01], p = 0.013), and Sex (β = -0.60, 95% CI [-1.04, -0.15], p = 0.009). The model explained 50.8% of the variance in ADL (R² = 0.508). For EQ-5D, significant predictors were MNA-SF score (β = -0.46, 95% CI [-0.56, -0.36], p = 0.000), Albumin (β = -0.12, 95% CI [-0.19, -0.05], p = 0.001), and BMI (β = 0.19, 95% CI [0.07, 0.32], p = 0.002). The model explained 41.3% of the variance in EQ-5D (R² = 0.413).

**Table 4:** Multiple linear regression models predicting ADL and EQ-5D scores in elderly inpatients.

Predictor	ADL Model	EQ-5D Model
	β (95% CI), p	β (95% CI), p
**MNA-SF (score)**	0.43 (0.34 to 0.53), p = 0.000	-0.46 (-0.56 to -0.36), p = 0.000
**Albumin (g/dL)**	0.13 (0.07 to 0.20), p = 0.000	-0.12 (-0.19 to -0.05), p = 0.001
**BMI (kg/m²)**	-0.09 (-0.21 to 0.02), p = 0.098	0.19 (0.07 to 0.32), p = 0.002
**Energy Intake (kcal/day)**	-0.00 (-0.00 to 0.00), p = 0.842	0.00 (-0.00 to 0.00), p = 0.127
**Age (years)**	-0.07 (-0.12 to -0.01), p = 0.013	0.04 (-0.02 to 0.10), p = 0.184
**Sex (Female vs. Male)**	-0.60 (-1.04 to -0.15), p = 0.009	0.29 (-0.20 to 0.79), p = 0.248
**R²**	0.508	0.413

Models were adjusted for age, sex, and comorbidities (e.g., hypertension, COPD). Coefficients (β), 95% confidence intervals (CI), and p-values are reported. MNA-SF: Mini Nutritional Assessment Short Form; BMI: Body Mass Index; ADL: Activities of Daily Living (modified Katz ADL index; range 0–7, higher = better function; functional impairment defined as score < 6); EQ-5D: EuroQol-5 Dimensions (range 5–15, higher = more health problems; higher scores represent worse health outcomes).

## Discussion

This cross-sectional study of 264 elderly inpatients at Military Hospital 354, Hanoi, Vietnam, identified a high prevalence of malnutrition: 34.8% were malnourished, and 37.9% were at risk, as determined by the Mini Nutritional Assessment Short Form (MNA-SF) (Table 2). These findings align with prior Vietnamese studies reporting 71.6-72.2% malnutrition or risk among elderly inpatients and global estimates ranging from 29% to 90% [1,2,4,10,14–16]. The high prevalence in our cohort, comparable to rates in Ethiopia (62.1%) and Uganda (33.3-52%) [16,17], underscores the pervasive burden of malnutrition in developing countries, where limited healthcare resources and inadequate nutritional screening exacerbate the problem [3,10,18].

The significant correlations between MNA-SF scores and serum Albumin (r = 0.44, p < 0.01) and Albumin and Activities of Daily Living (ADL) scores (r = 0.41, p < 0.01) highlight the interplay between nutritional status, biochemical profiles, and functional outcomes (Table 3). Hypoalbuminemia (<35 g/dL), observed in 33.0% of participants, was associated with lower ADL scores, consistent with studies linking low Albumin to functional decline, prolonged length of stay (LOS) (RR: 5.2), and mortality (RR: 4.1) [6,8,19]. Prealbumin, another marker, also predicts adverse outcomes in elderly inpatients; however, our study focused on Albumin due to its widespread clinical use [1]. Given the cross-sectional design, our findings reflect associations rather than causal relationships; for instance, hypoalbuminemia may result from malnutrition or contribute to it through bidirectional pathways of inflammation and nutrition. Regression analyses showed that MNA-SF and Albumin were significantly associated with ADL (β = 0.43, 95% CI [0.34, 0.53], p = 0.000; β = 0.13, 95% CI [0.07, 0.20], p = 0.000) and EuroQol-5 Dimensions (EQ-5D) (β = -0.46, 95% CI [-0.56, -0.36], p = 0.000; β = -0.12, 95% CI [-0.19, -0.05], p = 0.001), explaining 50.8% and 41.3% of variance, respectively (Table 4). These findings align with research linking nutritional status and biochemical markers to functional capacity and quality of life [2,5,15]. The R² values suggest a reasonable model fit; however, unmeasured factors, such as frailty or socioeconomic status, may also influence outcomes [4,20].

The BMI categorization (19- < 21, 21- < 23) for Asian populations, based on WHO recommendations, revealed nuanced differences, supporting its utility in identifying at-risk subgroups [11]. Socioeconomic factors, including low education and living alone, likely exacerbate malnutrition risk (OR: 1.83) [7], particularly in Vietnam, where there are limited hospital meals provisions and dysphagia (24.6%, OR: 3.21) pose additional challenges [10]. Inadequate dietary diversity, reported in Tanzania (70% of inpatients) [18], and low nutrient intake (e.g., protein, calcium) in Malaysia further parallel our findings on energy intake (950–2100 kcal/day), emphasizing the need for tailored nutritional interventions [21]. Although no significant sex differences were observed in albumin levels or energy intake (p > 0.05), the slight female predominance in functional impairment (ADL < 6: 35% females vs. 31% males) warrants further exploration in larger cohorts, potentially linked to gender-specific comorbidities, such as osteoporosis. Energy intake showed weaker correlations and non-significance in regression models, likely due to restricted dietary variation in the hospital setting, where meals are standardized and may not reflect pre-admission habits [21].

Regarding multicollinearity, although bivariate correlations between MNA-SF, BMI, and energy intake were high (r = 0.72-0.77), VIF values remained well below the conservative threshold of 3 (maximum VIF = 2.48 for MNA-SF; BMI: 2.31, energy intake: 1.13, Albumin: 1.24, age: 1.06, sex: 1.06), confirming that the regression models were not substantially affected by multicollinearity. Retaining all three nutritional indicators in the models is clinically justified, as MNA-SF captures overall nutritional risk through a composite screening tool, BMI reflects anthropometric status, and energy intake quantifies actual dietary consumption - each representing a distinct clinical dimension of nutritional assessment that contributes independently to understanding functional outcomes in this population.

### Clinical and policy implications

The high prevalence of malnutrition and its impact on functional outcomes suggest that routine screening with MNA-SF or Subjective Global Assessment (SGA) [15], combined with albumin monitoring, could facilitate early interventions to reduce LOS and improve recovery [1,2,16]. These findings directly support the implementation of hospital-based nutritional protocols outlined in Vietnam’s National Nutrition Strategy 2021–2030, such as mandating MNA-SF screening upon admission for elderly inpatients and integrating multidisciplinary nutritional support teams to address socioeconomic barriers, such as low income and dysphagia [22]. By operationalizing these strategies, hospitals can reduce malnutrition-related complications, aligning with national goals to enhance healthy aging and reduce healthcare inequities. Nutritional care processes, effective in improving intake among elderly inpatients [20], and texture-modified diets for dysphagic patients, as recommended in Vietnam [10], could improve outcomes. Addressing socioeconomic barriers, such as low income or social isolation, is crucial, as these factors significantly increase the risk of malnutrition [7]. Policy-level interventions, including hospital meal provision and nutrition education, outlined in Vietnam’s National Nutrition Strategy 2021–2030 [22], are essential to support Vietnam’s aging population [3]. Routine biochemical screening has been advocated since the 1970s and remains relevant for identifying at-risk elderly inpatients [23].

### Limitations

Several limitations warrant consideration. The cross-sectional design limits causal inferences between malnutrition, Albumin, and functional outcomes, necessitating longitudinal studies [10,20]. Single-center recruitment at Military Hospital 354, an urban tertiary facility, may introduce selection bias, as the sample may not represent rural or diverse Vietnamese populations, potentially limiting generalizability [4]. The 24-hour dietary recall may not accurately capture habitual intake due to intra-individual variability and recall bias; however, this limitation is mitigated by ward records. Unmeasured confounders, such as dysphagia severity, dietary preferences [8], or infection burden [9], may influence the explanatory power of the regression models (R² = 0.508 for ADL, 0.413 for EQ-5D). The summative EQ-5D dimension score (range 5–15) does not yield utility-weighted indices and therefore cannot be compared directly with most published EQ-5D-3L literature; its construct validity in the Vietnamese elderly population remains unestablished, and results should be interpreted as within-sample comparisons of cumulative problem burden rather than as health-state utilities.

### Future directions

Longitudinal studies are needed to establish causal relationships between nutritional status, biochemical markers, and functional outcomes in Vietnamese elderly inpatients [20]. Multi-center studies across Vietnam would enhance generalizability. Incorporating variables such as dysphagia screening (EAT-10) [10], mid-upper arm circumference (MUAC) [24], or frailty components could improve model fit [20]. Evaluating nutritional interventions, such as protein supplementation or dysphagia management [10,19], would inform evidence-based practices. Addressing socioeconomic determinants through community-based programs, as suggested by [7], could help reduce the risk of malnutrition among Vietnam’s aging population.

## Conclusion

This cross-sectional study of 264 elderly inpatients at Military Hospital 354, Hanoi, Vietnam, demonstrated a high prevalence of malnutrition: 34.8% were malnourished and 37.9% were at risk, as determined by the Mini Nutritional Assessment Short Form (MNA-SF). Hypoalbuminemia (<35 g/dL) affected 33.0% of participants, and 33.3% exhibited functional impairment in Activities of Daily Living (ADL). Significant correlations between MNA-SF scores, serum Albumin, and ADL (r = 0.41-0.63, p < 0.01), alongside regression models identifying MNA-SF and Albumin as predictors of ADL (β = 0.43 and 0.13, p < 0.01) and EuroQol-5 Dimensions (EQ-5D) (β = -0.46 and -0.12, p < 0.01), underscore the critical link between nutritional status, biochemical profiles, and functional outcomes (Tables 3, 4).

These findings underscore the urgent need for routine nutritional screening and interventions in Vietnamese hospitals to mitigate the impact of malnutrition on functional decline and quality of life, while acknowledging limitations, such as summative EQ-5D scoring, that may undervalue nuanced health utilities. By addressing these challenges through enhanced screening protocols, targeted nutritional support, and policy initiatives, Vietnam can improve health outcomes for its aging population. This study adds to the growing but still limited body of evidence on geriatric nutrition among elderly inpatients in developing countries, paving the way for future longitudinal research to inform evidence-based practices.

## Supporting information

S1 Checklist STROBE Checklist for cross-sectional studies. This file provides the completed STROBE checklist, which was used to guide the transparent reporting of the study.
(DOCX)
