# Supplementary material for: Nutritional intake, biochemical profiles, and functional outcomes in elderly inpatients: A hospital-based cross-sectional study in Vietnam
Source: PLOS Glob Public Health. 2026 Jul 16;6(7):e0006803. doi: 10.1371/journal.pgph.0006803 (PMC13374905; doi:10.1371/journal.pgph.0006803)
Supplement: S1 Checklist — This file provides the completed STROBE checklist, which was used to guide the transparent reporting of the study. (DOCX) [file pgph.0006803.s001.docx]

**STROBE Checklist for cross-sectional studies**

| **Item** | **Recommendation** | **Addressed in Manuscript** |
| --- | --- | --- |
|  | **Title and abstract** |  |
| 1a | Study design stated in title or abstract | Title includes “cross-sectional study” |
| 1b | Informative and balanced abstract | Abstract summarizes background, methods, results, and conclusions |
|  | **Introduction** |  |
| 2 | Scientific background and rationale | Provided in the Introduction, paragraphs 1–3 |
| 3 | Objectives and hypotheses | Clearly stated in the final paragraph of Introduction |
|  | **Methods** |  |
| 4 | Key elements of study design | Described in “Study design and setting” |
| 5 | Setting, locations, dates | Location and timeframe described (Military Hospital 354, 2024–2025) |
| 6a | Eligibility criteria, source of participants | Detailed in “Participants” section |
| 7 | Outcomes, exposures, confounders | Defined in “Data collection” and “Statistical analysis” |
| 8 | Sources and methods of assessment | Thoroughly described for each variable in “Data collection” |
| 9 | Efforts to address bias | Discussed in the “Limitations” section |
| 10 | Study size justification | Sample size and power calculation explained in “Participants” |
| 11 | Quantitative variable handling | Groupings and rationale described in “Statistical analysis” |
| 12a | Statistical methods and confounder control | Detailed in “Statistical analysis” |
| 12b | Methods for subgroups/interactions | Subgroup comparisons presented (e.g., MNA vs. BMI groups) |
| 12c | Missing data | Not explicitly addressed |
| 12d | Sampling strategy consideration | Not discussed; convenience sampling used |
| 12e | Sensitivity analyses | Not reported |
|  | **Results** |  |
| 13a | Numbers at each study stage | Total participants stated (n = 264) |
| 13b | Reasons for non-participation | Not mentioned |
| 13c | Flow diagram | Not included |
| 14a | Participant characteristics | Provided in Table 1 and 2 |
| 14b | Missing data per variable | Not reported |
| 15 | Outcome events or summaries | Reported in Tables 2–4 and Results section |
| 16a | Estimates, confidence intervals, adjustments | Regression models with 95% CI shown in Table 4 |
| 16b | Continuous variable categories | Clearly defined for BMI and MNA-SF |
| 16c | Relative to absolute risk | Not applicable |
| 17 | Additional analyses | Some subgroup analysis reported; no sensitivity analysis |
|  | **Discussion** |  |
| 18 | Key results summary | Stated in the first paragraph of Discussion |
| 19 | Study limitations | Discussed in “Limitations” section |
| 20 | Interpretation in context | Provided in “Discussion” and “Conclusion” |
| 21 | Generalisability | Addressed in “Limitations” and “Future directions” |
|  | **Other information** |  |
| 22 | Funding and role of funders | Declared as no specific funding |
