# Supplementary material for: Nutritional intake, biochemical profiles, and functional outcomes in elderly inpatients: A hospital-based cross-sectional study in Vietnam
Source: PLOS Glob Public Health. 2026 Jul 16;6(7):e0006803. doi: 10.1371/journal.pgph.0006803 (PMC13374905; doi:10.1371/journal.pgph.0006803)
Supplement: Attachment — Submitted filename: Response to Reviewers.docx [file pgph.0006803.s003.docx]

**Response to Reviewers**

**Manuscript title:** Nutritional intake, biochemical profiles, and functional outcomes in elderly inpatients: a hospital-based cross-sectional study in Vietnam

We sincerely thank the Academic Editor, Professor Razak Gyasi, and the reviewers for their thoughtful and constructive feedback. We have carefully revised the manuscript to address all concerns. Below, we provide a detailed, point-by-point response to each comment. All changes have been incorporated into the revised manuscript and clearly highlighted in the marked-up version.

**RESPONSE TO EDITORIAL REQUIREMENTS**

**1. Figure 1**

**Editor request:** Upload Figure 1 or remove all references.

**Response:** Figure 1 has been entirely removed from the manuscript. All references to a figure, including legends and related text, were deleted to ensure complete consistency.

**2. Figure 1 Legend**

**Response:** Since the figure was removed, no legend is included. This aligns with editorial instructions.

**3. Supporting Information Legends**

**Editor request:** Add a list of legends for Supporting Information files.

**Response:** Only the S1 Checklist (STROBE Checklist) is listed as Supporting Information. This ensures consistency between the uploaded files and the manuscript while meeting PLOS policy.

**4. Required Revisions to References**

**Response:** References have been reviewed, updated, and formatted in accordance with PLOS guidelines.

**RESPONSE TO REVIEWER #1**

**1. Novelty and Contribution**

**Comment:** Clarify what is unique about this study compared with other Asian research.

**Response:** We strengthened the Introduction to explicitly highlight the novelty of our study. We emphasize that this is one of the first Vietnamese hospital-based studies to integrate dietary intake, biochemical markers, and multiple functional outcomes (MNA-SF, ADL, EQ-5D). We now clearly articulate how the data address gaps relevant to geriatric nutrition policy in Vietnam.

**2. EQ-5D Scoring Method**

**Comment:** Provide justification and discuss limitations.

**Response:** A detailed justification has been added to the Methods section explaining the use of a summed EQ-5D dimension score due to the lack of validated Vietnamese population tariffs. A corresponding limitation is added to the Discussion.

**3. Multicollinearity Diagnostics**

**Comment:** Report whether VIF or other tests were performed.

**Response:** We added VIF diagnostics to the Methods and Results sections. All predictors had VIF < 3, indicating no concerning multicollinearity.

**4. Implausible Standard Deviation for Energy Intake**

**Comment:** SD was previously too large.

**Response:** We rechecked the dataset and corrected the error. The SD is now accurately reported as 174.88 kcal/day.

**5. Denominator Inconsistencies**

**Comment:** Ensure all Ns sum to 264 and are consistent across tables.

**Response:** We corrected all sample counts. MNA-SF categories now total 259, and we have added a footnote indicating that five participants had incomplete responses. EQ-5D group Ns now correctly match MNA categories.

**6. Causal Language**

**Comment:** Reframe causal statements due to cross-sectional design.

**Response:** We revised the Discussion to use non-causal, associative language throughout (e.g., “associated with,” “linked to”). All causal wording has been removed.

**7. Regression Interpretation**

**Comment:** Interpret regression results more cautiously.

**Response:** We added statements acknowledging the modest R² values and the potential influence of unmeasured factors.

**8. Dietary Recall Detail**

**Comment:** Add more detail on dietary assessment procedures.

**Response:** We expanded the Methods section to describe interviewer training, portion-size estimation tools, use of standardized models, and triangulation with hospital meal records.

**9. BMI Cutoff Rationale**

**Response:** We added justification referencing the WHO Expert Consultation (2004) and regional BMI standards.

**10. Shorten Discussion; Add Vietnam-Specific Implications**

**Response:** The Discussion has been refined, and we have added explicit references to Vietnam’s National Nutrition Strategy (2021–2030), identifying gaps in hospital nutrition policy.

**11. Reference Formatting**

**Response:** All references have been reviewed and updated to conform to PLOS formatting requirements.

**RESPONSE TO REVIEWER #2**

**Study Period**

**Comment:** Correct the inaccurate description of study dates.

**Response:** All dates have been corrected to January-June 2025 throughout the manuscript.

We sincerely thank the reviewers and editor for their insightful feedback, which has substantially improved our manuscript. We hope these revisions meet all expectations for acceptance.

We remain at your disposal for any further clarification.

Sincerely,

Le Xuan Hung

Corresponding author
