# Supplementary material for: Nutritional intake, biochemical profiles, and functional outcomes in elderly inpatients: A hospital-based cross-sectional study in Vietnam
Source: PLOS Glob Public Health. 2026 Jul 16;6(7):e0006803. doi: 10.1371/journal.pgph.0006803 (PMC13374905; doi:10.1371/journal.pgph.0006803)
Supplement: Attachment — Submitted filename: Response_to_Reviewers_auresp_2.docx [file pgph.0006803.s004.docx]

**Response to Reviewers**

**Manuscript title:** Nutritional intake, biochemical profiles, and functional outcomes in elderly inpatients: a hospital-based cross-sectional study in Vietnam

**Journal:** PLOS Global Public Health
**Manuscript ID:** PGPH-D-25-02136R1

Dear Professor Razak M. Gyasi,
Dear Reviewer,

We sincerely thank the Academic Editor and Reviewer #1 for their careful evaluation of our revised manuscript and for the constructive and encouraging comments. We are grateful for the recognition that the manuscript meets the scientific and ethical standards of PLOS Global Public Health.

In response, we have made minor editorial, clarificatory, and formatting refinements to enhance further clarity, transparency, and alignment with the journal's expectations. Below, we provide a point-by-point response to each comment. All changes have been incorporated into the revised manuscript and are highlighted in the tracked-changes version.

**RESPONSE TO REVIEWER #1**

**1. Conceptual framing of nutrition–biochemical–functional pathways**

**Comment:** The introduction would benefit from a more precise conceptual framing of how nutritional, biochemical, and functional parameters interrelate mechanistically.

**Response:** We have strengthened the Introduction by adding a concise mechanistic paragraph explicitly describing the inflammation–nutrition–albumin–function pathway. This addition explains how malnutrition may exacerbate inflammatory processes, leading to hypoalbuminemia, muscle wasting, and subsequent functional impairment (mobility, self-care, ADL).
This revision enhances conceptual coherence while maintaining an appropriate level of conciseness for a public health audience.

**2. National policy relevance**

**Comment:** The significance of the findings for national health policy could be more explicitly articulated.

**Response:** We expanded the Clinical and policy implications section to explicitly link our findings to Vietnam’s National Nutrition Strategy 2021–2030. We now specify how hospital-based nutritional screening (e.g., routine MNA-SF on admission) and multidisciplinary nutritional care could operationalize national policy goals related to healthy aging and equity in care.

**3. Sampling and generalizability**

**Comment:** The use of convenience sampling in a single tertiary facility should be explicitly acknowledged.

**Response:** We have revised the Limitations section to clearly state that the single-center, urban tertiary hospital setting and convenience sampling may limit the generalizability of the findings, particularly to rural or community-dwelling elderly populations.

**4. Dietary assessment limitations**

**Comment:** The 24-hour recall is prone to intra-individual variability and should be acknowledged as such.

**Response:** We explicitly note the limitations of a single 24-hour dietary recall, including intra-individual variability and potential recall bias, while also explaining that triangulation with ward meal records was used to partially mitigate this limitation.

**5. Regression modelling and non-significant energy intake**

**Comment:** Briefly explain why energy intake was non-significant in the regression models.

**Response:** We added an explanatory sentence in the Discussion noting that the energy intake may have been non-significant due to the restricted dietary variation in the hospital setting, where meals are relatively standardized and may not accurately reflect habitual pre-admission intake.

**6. Results presentation and EQ-5D interpretation**

**Comment:** Clarify that higher EQ-5D scores indicate worse health outcomes, thereby improving table clarity.

**Response:** We explicitly state in the text and the footnotes to Table 4 that higher EQ-5D scores represent worse health outcomes. In addition, Table 3 now highlights stronger correlations to improve readability. Minor rounding inconsistencies were corrected where appropriate.

**7. Causal inference**

**Comment:** Clarify that associations do not imply causality.

**Response:** We revised wording throughout the Discussion to consistently use non-causal language (e.g., “associated with,” “linked to”) and explicitly reiterate that the cross-sectional design does not allow causal inference.

**8. Policy implications: more concrete operationalization**
**Comment:** Tie findings more concretely to the Vietnam National Nutrition Strategy 2021–2030 and how hospital screening might operationalize MNA-SF.

**Response:** We expanded the policy section to provide concrete operational recommendations (e.g., MNA-SF screening on admission, multidisciplinary nutrition support, attention to dysphagia/texture-modified diets), explicitly framed within the National Nutrition Strategy 2021–2030.

**9. Gender differences**
**Comment:** A brief mention of gender differences would enhance nuance.

**Response:** We added a brief, cautious statement noting the slight female predominance in functional impairment in our sample and indicated that further research is required to explore possible gender-related drivers in larger cohorts.

**10. Limitations: completeness (including EQ-5D scoring) and reiteration in conclusion**
**Comment:** Ensure limitations cover cross-sectional design, single-center bias, 24-hour recall, and summative EQ-5D scoring; briefly note again in conclusion.

**Response:** We ensured that all these limitations are explicitly stated in the Limitations section. We also reiterate the summative EQ-5D scoring limitation in the Conclusion to maintain transparency and align with PLOS Global Public Health expectations.

**11. Timeframe formatting consistency (IMPORTANT)**
**Comment:** The data collection period should be formatted consistently (example given: “June 2024–April 2025”).

**Response:** We confirm that the **final, correct data collection period** for this study is **January to June 2025**, and we have ensured that this timeframe is stated consistently throughout the manuscript (Abstract and Methods). Any prior inconsistent date wording has been corrected for consistency.

**12. Minor proofreading and formatting**
**Comment:** Minor proofreading needed for spacing, capitalization, and date formatting.

**Response:** We performed a thorough proofread to correct minor spacing/capitalization issues, ensuring consistent date formatting and terminology throughout the manuscript.

We sincerely thank the Academic Editor and Reviewer #1 for their thoughtful and supportive evaluation. The revisions prompted by these comments have further improved the clarity, rigor, and policy relevance of our manuscript. We believe the revised version fully addresses all remaining concerns and is now suitable for publication in PLOS Global Public Health. We remain grateful for the opportunity to revise our work and are happy to provide any additional clarification if needed.

Sincerely,

Le Xuan Hung

Corresponding author
