# Supplementary material for: Nutritional intake, biochemical profiles, and functional outcomes in elderly inpatients: A hospital-based cross-sectional study in Vietnam
Source: PLOS Glob Public Health. 2026 Jul 16;6(7):e0006803. doi: 10.1371/journal.pgph.0006803 (PMC13374905; doi:10.1371/journal.pgph.0006803)
Supplement: Attachment — Submitted filename: Response_to_Reviewers_auresp_3.docx [file pgph.0006803.s005.docx]

**RESPONSE TO REVIEWER #3**

We sincerely thank Reviewer #3 for the careful re-evaluation of our manuscript and for the constructive comments that have helped improve methodological transparency and internal consistency. We have carefully addressed all points raised and revised the manuscript accordingly to ensure clarity, statistical rigor, and consistency with PLOS Global Public Health reporting standards. All changes have been incorporated into the revised manuscript and are highlighted in the tracked-changes version.

**1. Internal consistency of the study period**

**Comment:** The study period was inconsistently reported as January-December 2025 in the abstract and January-June 2025 in the manuscript body.

**Response:** We confirm that the correct study period is January to June 2025. The abstract has been revised to ensure full consistency with the Methods section. All references to the study timeframe have been carefully checked and standardized throughout the manuscript.

**2. MNA-SF category percentages do not sum to 100%**

**Comment:** Percentages of MNA-SF categories did not sum to 100%, suggesting inconsistency in denominators.

**Response:** We clarified that five participants had incomplete MNA-SF responses and were therefore excluded from category-specific calculations. Table 2 now includes a detailed footnote specifying that category percentages are calculated based on n=259 complete cases, while prevalence percentages reported in the text are based on the full sample (N=264). This approach maintains transparency and consistency with epidemiological reporting practice.

**3. Katz ADL scoring range clarification (0-6 vs 0-7)**

**Comment:** The Katz ADL scale is typically scored from 0-6; clarification was requested regarding the use of a modified version.

**Response:** We clarified in the Methods section that a modified Katz ADL index, comprising 7 activities, was used, with the addition of a mobility domain to better capture functional capacity among hospitalized older adults. The scoring range (0-7) and the definition of functional impairment (score <6) are now consistently reported across the Abstract, Methods, Results, and Table footnotes. Appropriate citation to the original Katz ADL instrument has been retained.

**4. Multicollinearity among nutritional indicators**

**Comment:** High correlations between MNA-SF, BMI, and energy intake raised concern about potential multicollinearity in regression models.

**Response:** We rechecked the regression diagnostics and now report detailed variance inflation factor (VIF) values for all predictors. VIF values ranged from 1.06 to 2.48, below both conventional (VIF<5) and conservative (VIF<3) thresholds, indicating no problematic multicollinearity. We also clarified that MNA-SF, BMI, and energy intake represent conceptually distinct dimensions of nutritional status (composite screening risk, anthropometric status, and dietary intake), justifying their simultaneous inclusion in the regression models. This explanation has been added to both the Statistical Analysis and Discussion sections.

**5. Interpretation of EQ-5D scoring approach**

**Comment:** The use of summed EQ-5D dimension scores instead of utility-weighted indices requires clarification.

**Response:** We clarified in the Methods and Limitations sections that the EQ-5D results are based on summed dimension scores (range 5-15) because Vietnamese EQ-5D value sets were not available at the time of analysis. We explicitly acknowledge that this approach does not produce utility-weighted health state indices and therefore limits comparability with standard EQ-5D studies. The results should therefore be interpreted as reflecting cumulative problem burden within the study population.

**6. Claim of “first empirical evidence.”**

**Comment:** The manuscript overstated novelty by claiming to provide the first empirical evidence from Vietnam.

**Response:** We revised the wording throughout the manuscript to avoid overstatement of novelty. The text now states that the study adds to the limited empirical evidence on nutritional and functional outcomes among hospitalized older adults in Vietnam, ensuring consistency with the cited literature.

**7. Convenience sampling and power calculation**

**Comment:** The use of a power calculation alongside convenience sampling required clarification.

**Response:** We clarified that the power calculation was conducted post hoc to assess sample adequacy, rather than to justify the sampling strategy. The Methods section now explicitly states the assumptions used (r=0.30, α=0.05, 80% power) and confirms that the achieved sample size exceeded the minimum required size, supporting the robustness of the statistical analyses.

**8. Data availability statement**

**Comment:** A clarification was requested regarding compliance with the PLOS data-sharing policy.

**Response:** We revised the Data Availability Statement to clarify that individual-level data cannot be publicly shared due to ethical restrictions imposed by the Institutional Review Board and limitations on participant consent. However, aggregate summary statistics underlying the findings are fully available within the manuscript tables and supporting files. Access to de-identified individual-level data may be granted upon reasonable request, subject to ethics approval.

We sincerely appreciate Reviewer #3’s careful and constructive evaluation. These revisions have improved the methodological clarity, transparency, and internal consistency of the manuscript. We believe the revised manuscript fully addresses all comments and meets the publication standards of PLOS Global Public Health.

Sincerely,

Le Xuan Hung
Corresponding author
