# Supplementary material for: Nutritional intake, biochemical profiles, and functional outcomes in elderly inpatients: A hospital-based cross-sectional study in Vietnam
Source: PLOS Glob Public Health. 2026 Jul 16;6(7):e0006803. doi: 10.1371/journal.pgph.0006803 (PMC13374905; doi:10.1371/journal.pgph.0006803)
Supplement: Peer review history

# Peer review: Decision Letter 0

**DOI:** 10.1371/journal.pgph.0006803.r001

**Authors:** Razak Gyasi

8 Oct 2025

PGPH-D-25-02136

Nutritional intake, biochemical profiles, and functional outcomes in elderly inpatients: a hospital-based cross-sectional study in Vietnam

PLOS Global Public Health

Dear Dr. Xuan Hung,

Thank you for submitting your manuscript to PLOS Global Public Health. After careful consideration, we feel that it has merit but does not fully meet PLOS Global Public Health’s publication criteria as it currently stands. Therefore, we invite you to submit a revised version of the manuscript that addresses the points raised during the review process.

Please submit your revised manuscript by Nov 22 2025 11:59PM. If you will need more time than this to complete your revisions, please reply to this message or contact the journal office at globalpubhealth@plos.org. When you're ready to submit your revision, log on to https://www.editorialmanager.com/pgph/ and select the 'Submissions Needing Revision' folder to locate your manuscript file.

Please include the following items when submitting your revised manuscript:

- A rebuttal letter that responds to each point raised by the editor and reviewer(s). You should upload this letter as a separate file labeled 'Response to Reviewers'.

- A marked-up copy of your manuscript that highlights changes made to the original version. You should upload this as a separate file labeled 'Revised Manuscript with Track Changes'.

- An unmarked version of your revised paper without tracked changes. You should upload this as a separate file labeled 'Manuscript'.

Guidelines for resubmitting your figure files are available below the reviewer comments at the end of this letter.

We look forward to receiving your revised manuscript.

Kind regards,

Prof Razak Gyasi, PhD, PD

Academic Editor

PLOS Global Public Health

Journal Requirements:

1. Please upload a copy of Figure 1 which you refer to in your text on page 18. Or if the figure is no longer to be included as part of the submission please remove all reference to it within the text.

Please provide separate figure files in .tif or .eps format and ensure that all files are under our size limit of 10MB.

For more information about how to convert your figure files please see our guidelines: https://journals.plos.org/globalpublichealth/s/figures

2. Please include a separate legend or caption for Figure 1 in your manuscript.

3. We have noticed that you have uploaded Supporting Information files, but you have not included a list of legends. Please add a full list of legends for your Supporting Information files before or after the references list.

4. If the reviewer comments include a recommendation to cite specific previously published works, please review and evaluate these publications to determine whether they are relevant and should be cited. There is no requirement to cite these works unless the editor has indicated otherwise.

Additional Editor Comments (if provided):

[Note: HTML markup is below. Please do not edit.]

Reviewers' comments:

Reviewer's Responses to Questions

**Comments to the Author**

1. Does this manuscript meet PLOS Global Public Health’s publication criteria? Is the manuscript technically sound, and do the data support the conclusions? The manuscript must describe methodologically and ethically rigorous research with conclusions that are appropriately drawn based on the data presented.

Reviewer #1: Yes

Reviewer #2: Yes

**********

2. Has the statistical analysis been performed appropriately and rigorously?

Reviewer #1: Yes

Reviewer #2: Yes

**********

3. Have the authors made all data underlying the findings in their manuscript fully available (please refer to the Data Availability Statement at the start of the manuscript PDF file)?

The PLOS Data policy requires authors to make all data underlying the findings described in their manuscript fully available without restriction, with rare exception. The data should be provided as part of the manuscript or its supporting information, or deposited to a public repository. For example, in addition to summary statistics, the data points behind means, medians and variance measures should be available. If there are restrictions on publicly sharing data—e.g. participant privacy or use of data from a third party—those must be specified.

Reviewer #1: Yes

Reviewer #2: Yes

**********

4. Is the manuscript presented in an intelligible fashion and written in standard English?

PLOS Global Public Health does not copyedit accepted manuscripts, so the language in submitted articles must be clear, correct, and unambiguous. Any typographical or grammatical errors should be corrected at revision, so please note any specific errors here.

Reviewer #1: Yes

Reviewer #2: Yes

**********

5. Review Comments to the Author

Please use the space provided to explain your answers to the questions above. You may also include additional comments for the author, including concerns about dual publication, research ethics, or publication ethics. (Please upload your review as an attachment if it exceeds 20,000 characters)

Reviewer #1: Dear Authors,

Thank you for submitting your manuscript entitled “Nutritional intake, biochemical profiles, and functional outcomes in elderly inpatients: a hospital-based cross-sectional study in Vietnam” to PLOS Global Public Health. We appreciate your important contribution to understanding malnutrition and functional outcomes among elderly inpatients in Vietnam.

After careful review, the manuscript was judged to be of interest but requiring major revision before it can be considered for publication. The main areas that require attention are:

Novelty and Contribution

Please clarify more explicitly what is unique about this study compared with existing research in Asia. Highlight its specific contribution to the Vietnamese context.

Methodological Clarifications

The EQ-5D scoring method (sum of raw scores 5–15) is non-standard. Please provide justification for this approach and acknowledge its limitations.

Regression models may suffer from multicollinearity between MNA-SF, BMI, and energy intake. Please indicate whether diagnostic tests (e.g., VIF) were performed and address implications.

Energy intake results (Table 2) report an implausibly high standard deviation. Please recheck and correct.

Denominators in some tables do not sum consistently to 264; please clarify.

Interpretation

Please reframe causal statements in the discussion, given the cross-sectional design.

Interpret regression results with more caution, given modest R² values.

Additional Revisions

Ensure consistency in reporting study dates (2024–2025 vs. 2025).

Clarify in the abstract and methods that higher EQ-5D scores indicate worse health.

Provide more detail on dietary recall procedures and rationale for BMI cutoffs.

Shorten the discussion by focusing more on Vietnam-specific implications, and where possible, reference local guidelines or policies.

Update references and ensure formatting is consistent with PLOS requirements.

We believe that with these revisions, your manuscript could make a valuable contribution to the literature on geriatric nutrition in low- and middle-income countries.

We therefore invite you to revise and resubmit your manuscript, addressing the comments above as well as those provided in the full reviewer reports.

Reviewer #2: This manuscript is written in a clear and understandable manner. It fills a gap in the scientific literature by indicating nutritional, biochemical markers and functional capacities to detect malnutrition in elderly patients and prevent the risk of prolonged hospitalization and mortality in a global context of aging population and increase of the elderly population. The only issue stands in the start and end dates of the study mentioned in the summary "January to december 2025".

**********

6. PLOS authors have the option to publish the peer review history of their article (what does this mean?). If published, this will include your full peer review and any attached files.

**Do you want your identity to be public for this peer review?**  If you choose “no”, your identity will remain anonymous but your review may still be made public.

For information about this choice, including consent withdrawal, please see our Privacy Policy.

Reviewer #1: No

Reviewer #2: No

**********

[NOTE: If reviewer comments were submitted as an attachment file, they will be attached to this email and accessible via the submission site. Please log into your account, locate the manuscript record, and check for the action link "View Attachments". If this link does not appear, there are no attachment files.]

-->Figure Resubmissions:

While revising your submission, we strongly recommend that you use PLOS’s NAAS tool (https://ngplosjournals.pagemajik.ai/artanalysis) to test your figure files. NAAS can convert your figure files to the TIFF file type and meet basic requirements (such as print size, resolution), or provide you with a report on issues that do not meet our requirements and that NAAS cannot fix.

After uploading your figures to PLOS’s NAAS tool - https://ngplosjournals.pagemajik.ai/artanalysis, NAAS will process the files provided and display the results in the "Uploaded Files" section of the page as the processing is complete. If the uploaded figures meet our requirements (or NAAS is able to fix the files to meet our requirements), the figure will be marked as "fixed" above. If NAAS is unable to fix the files, a red "failed" label will appear above. When NAAS has confirmed that the figure files meet our requirements, please download the file via the download option, and include these NAAS processed figure files when submitting your revised manuscript.

# Author comment: Author response to Decision Letter 1

**DOI:** 10.1371/journal.pgph.0006803.r002

22 Nov 2025

Attachment Submitted filename: Response to Reviewers.docx

# Peer review: Decision Letter 1

**DOI:** 10.1371/journal.pgph.0006803.r003

**Authors:** Razak Gyasi, Razak Gyasi

16 Dec 2025

PGPH-D-25-02136R1

Nutritional intake, biochemical profiles, and functional outcomes in elderly inpatients: a hospital-based cross-sectional study in Vietnam

PLOS Global Public Health

Dear Dr. Xuan Hung,

Thank you for submitting your manuscript to PLOS Global Public Health. After careful consideration, we feel that it has merit but does not fully meet PLOS Global Public Health’s publication criteria as it currently stands. Therefore, we invite you to submit a revised version of the manuscript that addresses the points raised during the review process.

Please submit your revised manuscript by Jan 15 2026 11:59PM. If you will need more time than this to complete your revisions, please reply to this message or contact the journal office at globalpubhealth@plos.org. When you're ready to submit your revision, log on to https://www.editorialmanager.com/pgph/ and select the 'Submissions Needing Revision' folder to locate your manuscript file.

Please include the following items when submitting your revised manuscript:

- A letter that responds to each point raised by the editor and reviewer(s). You should upload this letter as a separate file labeled 'Response to Reviewers'.
- A marked-up copy of your manuscript that highlights changes made to the original version. You should upload this as a separate file labeled 'Revised Manuscript with Track Changes'.
- An unmarked version of your revised paper without tracked changes. You should upload this as a separate file labeled 'Manuscript'.

Guidelines for resubmitting your figure files are available below the reviewer comments at the end of this letter.

We look forward to receiving your revised manuscript.

Kind regards,

Professor Razak M. Gyasi, PhD, PD

Academic Editor

PLOS Global Public Health

Journal Requirements:

1. If the reviewer comments include a recommendation to cite specific previously published works, please review and evaluate these publications to determine whether they are relevant and should be cited. There is no requirement to cite these works unless the editor has indicated otherwise.

2. Please review your reference list to ensure that it is complete and correct. If you have cited papers that have been retracted, please include the rationale for doing so in the manuscript text, or remove these references and replace them with relevant current references. Any changes to the reference list should be mentioned in the rebuttal letter that accompanies your revised manuscript. If you need to cite a retracted article, indicate the article’s retracted status in the References list and also include a citation and full reference for the retraction notice.

Additional Editor Comments (if provided):

[Note: HTML markup is below. Please do not edit.]

Reviewers' comments:

Reviewer's Responses to Questions

**Comments to the Author**

1. If the authors have adequately addressed your comments raised in a previous round of review and you feel that this manuscript is now acceptable for publication, you may indicate that here to bypass the “Comments to the Author” section, enter your conflict of interest statement in the “Confidential to Editor” section, and submit your "Accept" recommendation.

Reviewer #1: All comments have been addressed

**********

2. Does this manuscript meet PLOS Global Public Health’s publication criteria? Is the manuscript technically sound, and do the data support the conclusions? The manuscript must describe methodologically and ethically rigorous research with conclusions that are appropriately drawn based on the data presented.

Reviewer #1: Yes

**********

3. Has the statistical analysis been performed appropriately and rigorously?

Reviewer #1: Yes

**********

4. Have the authors made all data underlying the findings in their manuscript fully available (please refer to the Data Availability Statement at the start of the manuscript PDF file)?

The PLOS Data policy requires authors to make all data underlying the findings described in their manuscript fully available without restriction, with rare exception. The data should be provided as part of the manuscript or its supporting information, or deposited to a public repository. For example, in addition to summary statistics, the data points behind means, medians and variance measures should be available. If there are restrictions on publicly sharing data—e.g. participant privacy or use of data from a third party—those must be specified.

Reviewer #1: Yes

**********

5. Is the manuscript presented in an intelligible fashion and written in standard English?

PLOS Global Public Health does not copyedit accepted manuscripts, so the language in submitted articles must be clear, correct, and unambiguous. Any typographical or grammatical errors should be corrected at revision, so please note any specific errors here.

Reviewer #1: Yes

**********

6. Review Comments to the Author

Please use the space provided to explain your answers to the questions above. You may also include additional comments for the author, including concerns about dual publication, research ethics, or publication ethics. (Please upload your review as an attachment if it exceeds 20,000 characters)

Reviewer #1: Title: Nutritional intake, biochemical profiles, and functional outcomes in elderly inpatients: a hospital-based cross-sectional study in Vietnam.

1. Significance and Relevance

This study addresses an increasingly critical public health issue in low- and middle-income countries: malnutrition among elderly inpatients and its association with functional outcomes and biochemical markers. The focus on Vietnam adds important regional data to an underexplored context. The study is timely, clinically relevant, and aligned with the WHO Decade of Healthy Ageing and PLOS Global Public Health’s mission to highlight inequities in global health.

Strengths

Original data from a developing country context where geriatric malnutrition is poorly documented.

Integration of biochemical (albumin), anthropometric (BMI), and functional measures (ADL, EQ-5D).

Ethical conduct and transparent methodology.

Areas for Improvement

The introduction would benefit from a slightly clearer conceptual framing of how nutritional, biochemical, and functional parameters interrelate mechanistically (e.g., inflammation–nutrition–function pathways).

The significance of the findings for national health policy could be more explicitly articulated in the discussion.

2. Methods

The revised manuscript presents a clearer and more rigorous description of study design, inclusion criteria, and data analysis compared with the initial submission.

Strengths

Well-described ethical process, inclusion/exclusion criteria, and quality control in data collection.

Use of validated tools (MNA-SF, ADL, EQ-5D) and appropriate statistical methods (Spearman correlations, multiple regression).

Consideration of potential confounders (age, sex, comorbidities).

Weaknesses and Recommendations

Sampling and Generalizability: The use of convenience sampling in a single tertiary facility introduces potential bias. The authors should explicitly acknowledge this and state that findings may not generalize to community or rural populations.

Dietary Assessment: The 24-hour recall is standard but prone to intra-individual variability; mention of this limitation would strengthen transparency.

Regression Modelling: The authors appropriately checked variance inflation factors (VIF <3), but should briefly explain why energy intake was non-significant—possibly due to restricted dietary variation in hospital settings.

Timeframe Clarification: In the revised version, the data collection period (June 2024–April 2025) is now clear, but should be formatted consistently (e.g., “June 2024 to April 2025”).

Overall, methodological rigor is sound and appropriate for publication after minor edits.

3. Results

The results are statistically coherent and presented with clarity. Tables are logically structured and readable.

Highlights

The prevalence of malnutrition (34.8%) and hypoalbuminemia (33%) are well contextualized.

Correlation patterns (MNA–Albumin, MNA–ADL) are consistent with international evidence.

Regression outputs are complete and interpretable.

Areas to Refine

Table 3’s correlation matrix is dense; consider shading or formatting for clarity.

Table 4’s regression results: explicitly note that “higher EQ-5D scores represent worse health outcomes.”

Slight rounding inconsistencies remain (e.g., ADL β coefficients could be given to two decimals).

4. Discussion and Interpretation

The revised discussion is notably stronger, better integrated with current literature, and more reflective in tone.

Strengths

Effective comparison with previous Vietnamese and regional studies.

Balanced acknowledgement of the high malnutrition prevalence and its implications for hospital practice.

Discussion of biochemical and functional interplay is evidence-based.

Remaining Suggestions

Clarify the causal inference limitations: emphasize that associations do not imply directionality.

The policy implications section could more concretely tie findings to the Vietnam National Nutrition Strategy 2021–2030, specifying how hospital screening protocols might operationalize MNA-SF use.

A brief mention of gender differences (given 50/50 sex distribution) would enhance nuance.

5. Limitations

The authors have substantially improved this section.

To make it fully compliant with PLOS Global Public Health standards, ensure it explicitly covers:

Cross-sectional design (no causality).

Single-center bias.

24-hour recall limitations.

Summative EQ-5D scoring (acknowledged, but worth briefly noting again in the conclusion).

6. Ethical and Data Transparency

Ethical approval, consent procedures, and IRB details are comprehensive.

Data availability statement is appropriate given local restrictions.

Declaration of AI use (for grammar editing only) aligns with PLOS policy.

No ethical or integrity concerns are identified.

7. Presentation and Language

The revised manuscript reads fluently, and the English is suitable for publication.

Minor proofreading is needed for spacing, capitalization (“likesuch as Vietnam”), and consistent date formatting.

Figures and tables are well labeled and suitable for print.

8. Overall Assessment

This revision demonstrates significant improvement in clarity, coherence, and methodological transparency. The manuscript meets the scientific validity and ethical standards expected for PLOS Global Public Health.

Major Strengths After Revision

Comprehensive analytical integration of nutritional, biochemical, and functional parameters.

Contextually rich and globally relevant findings.

Clear and responsible interpretation of cross-sectional data.

Minor Weaknesses Remaining

Slight residual editorial issues and formatting inconsistencies.

Minor elaboration needed on generalizability and mechanistic pathways.

9. Recommendation

Decision: Minor Revision (Accept after language and presentation refinements)

Justification:

The revised manuscript is methodologically robust and addresses prior review concerns. It makes a meaningful contribution to the literature on geriatric nutrition and health outcomes in low- and middle-income settings. Final editorial and stylistic adjustments will prepare it for publication.

**********

7. PLOS authors have the option to publish the peer review history of their article (what does this mean?). If published, this will include your full peer review and any attached files.

**Do you want your identity to be public for this peer review?**  If you choose “no”, your identity will remain anonymous but your review may still be made public.

For information about this choice, including consent withdrawal, please see our Privacy Policy.

Reviewer #1: **Yes:** Ibrahim A. Abdulganiyyu

**********

[NOTE: If reviewer comments were submitted as an attachment file, they will be attached to this email and accessible via the submission site. Please log into your account, locate the manuscript record, and check for the action link "View Attachments". If this link does not appear, there are no attachment files.]

Figure Resubmissions:

While revising your submission, we strongly recommend that you use PLOS’s NAAS tool (https://ngplosjournals.pagemajik.ai/artanalysis) to test your figure files. NAAS can convert your figure files to the TIFF file type and meet basic requirements (such as print size, resolution), or provide you with a report on issues that do not meet our requirements and that NAAS cannot fix.

After uploading your figures to PLOS’s NAAS tool - https://ngplosjournals.pagemajik.ai/artanalysis, NAAS will process the files provided and display the results in the "Uploaded Files" section of the page as the processing is complete. If the uploaded figures meet our requirements (or NAAS is able to fix the files to meet our requirements), the figure will be marked as "fixed" above. If NAAS is unable to fix the files, a red "failed" label will appear above. When NAAS has confirmed that the figure files meet our requirements, please download the file via the download option, and include these NAAS processed figure files when submitting your revised manuscript.

# Author comment: Author response to Decision Letter 2

**DOI:** 10.1371/journal.pgph.0006803.r004

7 Jan 2026

Attachment Submitted filename: Response_to_Reviewers_auresp_2.docx

# Peer review: Decision Letter 2

**DOI:** 10.1371/journal.pgph.0006803.r005

**Authors:** Razak Gyasi, Razak Gyasi, Alejandro Torrado Pacheco

12 Mar 2026

PGPH-D-25-02136R2

Nutritional intake, biochemical profiles, and functional outcomes in elderly inpatients: a hospital-based cross-sectional study in Vietnam

PLOS Global Public Health

Dear Dr. Xuan Hung,

Thank you for submitting your manuscript to PLOS Global Public Health. After careful consideration, we feel that it has merit but does not fully meet PLOS Global Public Health’s publication criteria as it currently stands. Therefore, we invite you to submit a revised version of the manuscript that addresses the points raised during the review process.

The manuscript has been evaluated by a third reviewer and their comments are available below. They request several clarifications, especially on the study methodology. Please address all comments carefully.

Please submit your revised manuscript by Apr 25 2026 11:59PM. If you will need more time than this to complete your revisions, please reply to this message or contact the journal office at globalpubhealth@plos.org. When you're ready to submit your revision, log on to https://www.editorialmanager.com/pgph/ and select the 'Submissions Needing Revision' folder to locate your manuscript file.

Please include the following items when submitting your revised manuscript:

- A letter that responds to each point raised by the editor and reviewer(s). You should upload this letter as a separate file labeled 'Response to Reviewers'.
- A marked-up copy of your manuscript that highlights changes made to the original version. You should upload this as a separate file labeled 'Revised Manuscript with Track Changes'.
- An unmarked version of your revised paper without tracked changes. You should upload this as a separate file labeled 'Manuscript'.

Guidelines for resubmitting your figure files are available below the reviewer comments at the end of this letter.

We look forward to receiving your revised manuscript.

Kind regards,

Alejandro Torrado Pacheco, PhD

PLOS Editor

PLOS Global Public Health

Journal Requirements:

If the reviewer comments include a recommendation to cite specific previously published works, please review and evaluate these publications to determine whether they are relevant and should be cited. There is no requirement to cite these works unless the editor has indicated otherwise.

Additional Editor Comments (if provided):

[Note: HTML markup is below. Please do not edit.]

Reviewers' comments:

Reviewer's Responses to Questions

**Comments to the Author**

1. If the authors have adequately addressed your comments raised in a previous round of review and you feel that this manuscript is now acceptable for publication, you may indicate that here to bypass the “Comments to the Author” section, enter your conflict of interest statement in the “Confidential to Editor” section, and submit your "Accept" recommendation.

Reviewer #3: All comments have been addressed

**********

2. Does this manuscript meet PLOS Global Public Health’s publication criteria? Is the manuscript technically sound, and do the data support the conclusions? The manuscript must describe methodologically and ethically rigorous research with conclusions that are appropriately drawn based on the data presented.

Reviewer #3: Partly

**********

3. Has the statistical analysis been performed appropriately and rigorously?

Reviewer #3: No

**********

4. Have the authors made all data underlying the findings in their manuscript fully available (please refer to the Data Availability Statement at the start of the manuscript PDF file)?

The PLOS Data policy requires authors to make all data underlying the findings described in their manuscript fully available without restriction, with rare exception. The data should be provided as part of the manuscript or its supporting information, or deposited to a public repository. For example, in addition to summary statistics, the data points behind means, medians and variance measures should be available. If there are restrictions on publicly sharing data—e.g. participant privacy or use of data from a third party—those must be specified.

Reviewer #3: Yes

**********

5. Is the manuscript presented in an intelligible fashion and written in standard English?

PLOS Global Public Health does not copyedit accepted manuscripts, so the language in submitted articles must be clear, correct, and unambiguous. Any typographical or grammatical errors should be corrected at revision, so please note any specific errors here.

Reviewer #3: Yes

**********

6. Review Comments to the Author

Please use the space provided to explain your answers to the questions above. You may also include additional comments for the author, including concerns about dual publication, research ethics, or publication ethics. (Please upload your review as an attachment if it exceeds 20,000 characters)

Reviewer #3: 1. Internal inconsistencies in key data

There are notable discrepancies between the abstract/cover page and the manuscript body that undermine confidence in data integrity:

The abstract (cover page) states data collection ran from "January to December 2025," while the manuscript body states "January to June 2025." The response to reviewers confirms January–June 2025, but this inconsistency was not corrected in the submission abstract.

MNA-SF category percentages do not sum to 100%: malnourished (34.8%) + at risk (37.9%) + normal (25.4%) = 98.1%, with five participants excluded from category analysis. This should be explicitly reconciled in Table 2 and the text. The cover-page abstract states "33.0% had hypoalbuminemia" but also that 37.9% were "at risk (8-11)" — both figures appear inconsistently across versions of the abstract.

The Katz ADL scale is described as ranging from 0–7, yet standard Katz scoring yields 0–6. The authors should clarify whether they used a modified version and cite accordingly.

2. Multicollinearity concern not adequately addressed

Table 3 reveals very high correlations between MNA-SF and BMI (r = 0.72) and MNA-SF and energy intake (r = 0.77), and between BMI and energy intake (r = 0.74). These are strong enough to raise serious multicollinearity concerns in the regression models. The reported VIF values (MNA-SF: 2.1, BMI: 1.8, energy intake: 2.4) appear implausibly low given these inter-correlations. The authors should recheck and report VIF values transparently, or justify statistically why multicollinearity is not a concern here.

3. Cross-sectional design overstated as "first empirical evidence"

The manuscript claims to provide "the first empirical evidence from Vietnam linking nutritional and functional measures among elderly inpatients." This is an overclaim — the authors themselves cite Vietnamese studies (references 4 and 8) that examine malnutrition and functional outcomes in similar populations. This claim should be moderated.

4. EQ-5D scoring approach requires stronger justification

The summative scoring of EQ-5D (range 5–15) is non-standard and methodologically problematic. The EQ-5D-3L was designed to generate health utility index scores (via value sets), not summative dimension scores. While the authors acknowledge this limitation, the approach limits the clinical interpretability and comparability of findings. The manuscript should more explicitly state that the EQ-5D results cannot be compared to most published literature and that the construct validity of this summative approach in the Vietnamese elderly population has not been established.

5. Convenience sampling and power calculation inconsistency

The authors describe a "convenience sample" of 264 participants, yet also present a power calculation. A power calculation is typically used for probability-based sampling to ensure adequate sample size — its use with convenience sampling should be better justified or reframed. Furthermore, the power calculation parameters (detecting r = 0.30, 80% power, α = 0.05, 10% attrition) are stated but not shown. The minimum required sample size derived from these parameters should be explicitly reported.

**********

7. PLOS authors have the option to publish the peer review history of their article (what does this mean?). If published, this will include your full peer review and any attached files.

**Do you want your identity to be public for this peer review?**  If you choose “no”, your identity will remain anonymous but your review may still be made public.

For information about this choice, including consent withdrawal, please see our Privacy Policy.

Reviewer #3: No

**********

[NOTE: If reviewer comments were submitted as an attachment file, they will be attached to this email and accessible via the submission site. Please log into your account, locate the manuscript record, and check for the action link "View Attachments". If this link does not appear, there are no attachment files.]

-->Figure Resubmissions:

While revising your submission, we strongly recommend that you use PLOS’s NAAS tool (https://ngplosjournals.pagemajik.ai/artanalysis) to test your figure files. NAAS can convert your figure files to the TIFF file type and meet basic requirements (such as print size, resolution), or provide you with a report on issues that do not meet our requirements and that NAAS cannot fix.

After uploading your figures to PLOS’s NAAS tool - https://ngplosjournals.pagemajik.ai/artanalysis, NAAS will process the files provided and display the results in the "Uploaded Files" section of the page as the processing is complete. If the uploaded figures meet our requirements (or NAAS is able to fix the files to meet our requirements), the figure will be marked as "fixed" above. If NAAS is unable to fix the files, a red "failed" label will appear above. When NAAS has confirmed that the figure files meet our requirements, please download the file via the download option, and include these NAAS processed figure files when submitting your revised manuscript.

# Author comment: Author response to Decision Letter 3

**DOI:** 10.1371/journal.pgph.0006803.r006

18 Apr 2026

Attachment Submitted filename: Response_to_Reviewers_auresp_3.docx

# Editor report: Decision Letter 3

**DOI:** 10.1371/journal.pgph.0006803.r007

**Authors:** Razak Gyasi, Razak Gyasi, Alejandro Torrado Pacheco, Razak Gyasi

18 Jun 2026

Nutritional intake, biochemical profiles, and functional outcomes in elderly inpatients: a hospital-based cross-sectional study in Vietnam

PGPH-D-25-02136R3

Dear Mr. Xuan Hung,

We are pleased to inform you that your manuscript 'Nutritional intake, biochemical profiles, and functional outcomes in elderly inpatients: a hospital-based cross-sectional study in Vietnam' has been provisionally accepted for publication in PLOS Global Public Health.

Before your manuscript can be formally accepted you will need to complete some formatting changes, which you will receive in a follow up email. A member of our team will be in touch with a set of requests.

Please note that your manuscript will not be scheduled for publication until you have made the required changes, so a swift response is appreciated.

IMPORTANT: The editorial review process is now complete. PLOS will only permit corrections to spelling, formatting or significant scientific errors from this point onwards. Requests for major changes, or any which affect the scientific understanding of your work, will cause delays to the publication date of your manuscript.

If your institution or institutions have a press office, please notify them about your upcoming paper to help maximize its impact. If they'll be preparing press materials, please inform our press team as soon as possible -- no later than 48 hours after receiving the formal acceptance. Your manuscript will remain under strict press embargo until 2 pm Eastern Time on the date of publication. For more information, please contact globalpubhealth@plos.org.

Thank you again for supporting Open Access publishing; we are looking forward to publishing your work in PLOS Global Public Health.

Best regards,

Prof Razak Gyasi, PhD, PD

Academic Editor

PLOS Global Public Health

Reviewer Comments (if any, and for reference):
